# The Effects of Periodontal Treatment on Psoriasis: A Systematic Review of Limited Clinical and Preclinical Evidence

**DOI:** 10.3390/jcm15062434

**Published:** 2026-03-22

**Authors:** Daniela Cornelia Condor, Ana-Maria Copaciu-Condor, Andreea Kui, Marius Negucioiu, Smaranda Buduru, Ondine Patricia Lucaciu

**Affiliations:** 1 Periodontology Discipline, Department 3—Oral Rehabilitation, Faculty of Dental Medicine, “Iuliu Hațieganu” University of Medicine and Pharmacy, 400006 Cluj-Napoca, Romania; cornelia.condor@umfcluj.ro; 2 Prosthodontics Discipline, Department 4—Prosthodontics and Dental Materials, Faculty of Dental Medicine, “Iuliu Hațieganu” University of Medicine and Pharmacy, 400006 Cluj-Napoca, Romania; 3 Oral Health Discipline, Department 3—Oral Rehabilitation, Faculty of Dental Medicine, “Iuliu Hațieganu” University of Medicine and Pharmacy, 400006 Cluj-Napoca, Romania

**Keywords:** periodontitis, non-surgical periodontal treatment, psoriasis, palmoplantar pustulosis, systematic review

## Abstract

**Background/Objectives**: Psoriasis and periodontitis share inflammatory pathways. Current evidence suggests a bidirectional non-causal relation. However, the evidence on the effects of periodontal treatment on psoriasis outcomes (severity, inflammatory markers, quality of life) is limited. This study aims to synthetize the available clinical and preclinical evidence of periodontal treatment effects on psoriasis outcomes, in patients with comorbid psoriasis and periodontitis (CRD420261298145). **Methods**: Several databases (PubMed, WebOfScience, ScienceDirect, ProQuest and GoogleScholar) were searched for relevant articles, without language or time restrictions. We included randomised and non-randomised clinical studies on humans, and controlled animal experiments. Interventions included periodontal treatment (surgical and non-surgical). Outcomes were the Psoriasis Area and Severity Index and dermatology-specific quality of life scores; secondary outcomes included inflammatory biomarkers and periodontal parameters. Studies were screened in duplicate, data extracted independently and risk of bias was assessed using Cochrane RoB 2, ROBINS I, NOS and SYRCLE. **Results**: A total of five studies were included in this systematic review (four clinical studies and one preclinical studies). Three studies directly assessed post-treatment psoriasis outcomes, with two studies investigating inflammation mediators as secondary outcomes. Two studies directly assessed PASI (Psoriasis Area and Severity Index) modifications, both studies confirming PASI scores decreasing post-periodontal treatment; one study also reported DLQI (Dermatology Life Quality Index). Typical follow-up durations ranged from 8 to 10 weeks for interventional studies, to 5 years for one cohort study. **Conclusions:** Although momentarily limited by the small number of available studies, the results of this review suggest that periodontal treatment may be associated with improvements in psoriasis outcomes. Further studies on larger samples, with longer follow-up periods would be necessary to confirm and possibly strengthen the existing results.

## 1. Introduction

Psoriasis is a chronic, immune-mediated dermatosis, characterised by exacerbation-relapse courses. It mainly affects the skin and joints. The main manifestation of psoriasis is demarcated erythematous plaques covered in silver-coloured scales, which significantly impact patients’ quality of life, causing physical and psychological stress. Psoriasis affects approximately 2–3% of the global population (125 million individuals), but prevalence varies with region and race [1,2,3]. Psoriatic lesions are often painful, burning or itchy; abnormal generation of keratinocytes leads to an accelerated cycle of cell desquamation and epidermal hyperplasia. The existence of an immune-mediated mechanism in psoriasis development is proven by the presence of neutrophilic infiltrate in both epidermis and dermis, as a consequence of the presence of elevated pro-inflammatory cytokines and inflammatory mediators (IL-17A, TNF-α, and IL-36) [4]. Th1/Th17 T-helper cells and dendritic cells are central to the manifestation of psoriasis, their excessive activation, differentiation and cytokine production driving the inflammatory cycle [1]. Psoriasis aetiology is multifactorial, with genetic predisposition and environmental factors contributing to disease development, including obesity, smoking and excessive alcohol consumption [5]. Microbial dysbiosis has been linked to psoriasis development as well [6].

The multifactorial aetiology, microbial dysbiosis, and exacerbated inflammatory response involving the Th17-axis are all shared characteristics of psoriasis and periodontitis [1,7,8]; periodontitis is much more prevalent, affecting more than 1 billion adults worldwide [9]. Periodontitis is considered a chronic, multifactorial disease caused by aberrant inflammatory immune-mediated responses, triggered by dysbiotic oral biofilms and exacerbated by local and systemic factors [10,11]. Furthermore, periodontitis has been associated with increased risks of systemic diseases like cardiovascular disease, diabetes, cancer and overall mortality. Systemic inflammation triggered by circulating periodontopathogens (bacterial translocation) has been cited as a possible mechanism linking periodontitis to systemic pathologies [12,13]. A recent meta-analysis found that individuals with periodontitis had a 2.87-fold increased risk of psoriasis compared to periodontally healthy patients, showing a robust association between the two [14]. Available evidence suggests the opposite is true: patients with psoriasis show poorer periodontal status, and periodontitis prevalence is increased [15,16,17,18,19].

**Figure 1:**
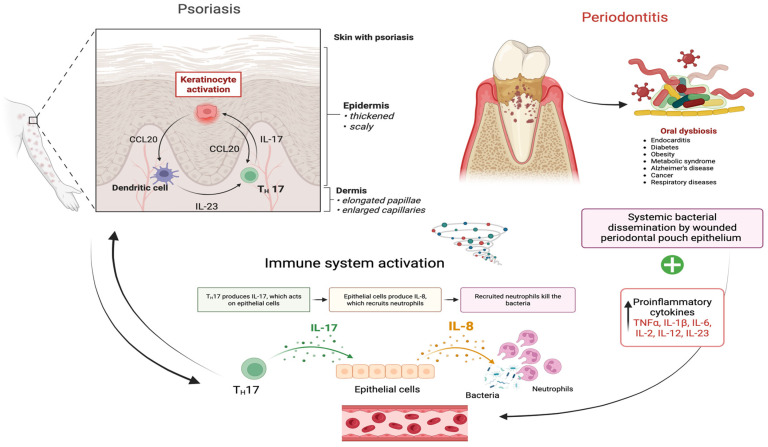
The relation between periodontitis, psoriasis and the immune system. Dysbiotic plaque bacteria in periodontal pockets activate innate immune cells, triggering release of IL-1β, IL-6, TNF-α into systemic circulation. These mediators activate the IL-23/Th17/TNF-α axis, driving production of IL-17A/F and TNF-α, which may amplify psoriatic skin inflammation through keratinocyte activation and immune cell recruitment. Psoriasis also amplifies systemic inflammation through activation of the same cytokines. The chronic inflammation status is maintained through both mechanisms. Created in BioRender. Copaciu-Condor, A. (2026) https://BioRender.com/etyriiz (accessed on 13 March 2026).

Despite the confirmed strong bidirectional association, the relationship between periodontitis and psoriasis has not been established as causal [20]. Currently, the connection between psoriasis and periodontitis is based on observational data, and while this suggests a promising direction to explore in research to reveal further mechanistic evidence, it does not imply a therapeutic relation. Since no clear causality has been consensually demonstrated, very few studies highlight the impact of treatment of one disease on the severity of the other. The limited number of existing studies are hypothesis-driven and provide preliminary evidence. One hypothesis states that since periodontal treatment has been proven to significantly lower levels of circulating inflammatory cytokines (including Th17-axis cytokines (IL-17A, IL-21, IL-22, IL-23) (Figure 1), this anti-inflammatory effect may extend dermatological benefits, providing an effective adjuvant therapy [21].

Evaluating the limited existing evidence on the effects of a disease-specific intervention (periodontal treatment) on the disease-specific outcomes of psoriasis is important because it fills a distinct knowledge gap beyond observational studies on prevalence or bidirectional risk. Previous systematic reviews focus on prevalence and association, and no systematic review assessed the treatment effect of periodontitis on psoriasis outcomes [10,15,19,22].

The objective of this study was to systematically evaluate the effects of periodontal treatment (surgical and non-surgical) on psoriasis severity, manifestations, quality of life and inflammatory biomarkers in adults with comorbid psoriasis and periodontitis, using both human clinical trials and pre-clinical animal intervention studies. Secondary objectives included identifying mechanistic evidence of periodontal treatment effects on psoriasis using pre-clinical evidence and identifying existing research gaps for further study protocols development.

## 2. Materials and Methods

### 2.1. Protocol and Registration

This review used the Preferred Reporting Items for Systematic Reviews and Meta-Analyses (PRISMA) 2020 reporting guidelines for both the study design and the abstract [23]. PRISMA checklist is available in Supplementary Materials (Supplementary File S1). This review was registered at the International Prospective Register of Systematic Reviews (PROSPERO number CRD420261298145).

### 2.2. Study Design and Eligibility Criteria

This review was designed to answer the following questions: “Does periodontal treatment result in clinically meaningful improvements in psoriasis severity, inflammatory markers or quality of life compared to no treatment in patients with comorbid psoriasis and periodontitis?”; “In animal models with experimental periodontitis and psoriasis, will periodontal therapy simulation result in clinically meaningful improvements in psoriasis-relevant outcomes and/or periodontal outcomes, compared with no treatment or single-disease controls?”

The inclusion and exclusion criteria of this systematic review were organised according to the PECOS strategy. Clinical and pre-clinical studies criteria were elaborated separately.

#### 2.2.1. Inclusion Criteria

Clinical studies:

- P (population) = adult human patients, with diagnosed psoriatic disease (any severity, any subtype: plaque, guttate, pustular, erythrodermic; including psoriatic arthritis and palmo-plantar pustulosis) and diagnosed periodontitis (any stage/grade per AAP/EFP 2017 classification, or equivalent historical definitions); studies on patients without comorbid psoriasis and periodontitis were included if comorbidity was developed during study follow-up period;
- E (exposure) = surgical or non-surgical periodontal treatment (including systemic or local antimicrobial adjuvant therapy) and/or maintenance phase therapy and prophylaxis;
- C (control) = no periodontal treatment, standard dermatological care only, baseline or pre-treatment status;
- O (type of outcome measures) = psoriasis-related outcomes objectively determined before-and-after periodontal treatment, including: psoriasis severity—PASI (Psoriasis Area and Severity Index) changes reported in absolute points (when available), PASI response rates, BSA (body surface area) affected by psoriasis, time to psoriasis flare or relapse, psoriasis incidence (in patients without comorbidity), DLQI (Dermatology Life Quality Index) with a minimal clinically important difference (MCID) value set to 4 points, periodontal parameters (including but not limited to probing depth (PD), clinical attachment loss (CAL), bleeding on probing (BOP), plaque index (PI), radiographic bone loss), inflammatory and immunological biomarkers from serum or saliva or gingival crevicular fluid (including but not limited to IL-6, IL-17, IL-23, TNF-α, CRP);
- S (type of studies) = randomised and non-randomised controlled trials, prospective studies with minimum follow-up of 4 weeks post-intervention, retrospective studies, cohort studies.

Preclinical studies:

- P = rodent models or other animal models with experimental periodontitis and experimental psoriasis (e.g., ligature-induced periodontitis + imiquimod (IMQ)-induced psoriasis);
- I = periodontal therapy simulation (ligature removal, debridement), pharmacological periodontal interventions, combined systemic periodontal and psoriasis treatment;
- C = disease controls with no periodontal treatment, sham controls, single-disease controls;
- O = psoriasis relevant outcomes including but not limited to epidermal thickness (histomorphometry), dermal inflammatory infiltrate, skin lesions size and/or severity, systemic inflammatory markers; periodontal outcomes.

#### 2.2.2. Exclusion Criteria

Clinical studies:

- P = paediatric populations;
- I = studies examining only periodontal status without periodontal intervention;
- O = studies with insufficient or non-relevant outcome data (studies without psoriasis-relevant outcomes);
- S = case reports, case series, cross-sectional studies without pre-post intervention assessment, cohort studies without prospective component, review studies and meta-analyses, editorials, conference abstracts without full data available, correspondence, in vitro studies;
- Preclinical studies: descriptive/observational animal studies with no interventions, single-disease models, studies not reporting psoriasis and/or periodontal outcomes.

### 2.3. Search Strategy and Study Selection Process

An electronic search was conducted until February 2026 in the following databases: PubMed, ProQuest, Web of Science, ScienceDirect. Grey literature was searched using Google Scholar (the first 10 results pages were considered). No date or language restrictions were applied. Additionally, manual reference searching (“snowballing”) was conducted for theme-related systematic reviews and identified relevant studies. The electronic literature search was done by two independent researchers. The search consisted of keywords (such as Medical Subject Headings or MeSH) for psoriasis, periodontitis, periodontal treatment, and animal models, combined with Boolean operators “AND” and “OR”, as well as keyword searching of title, abstract and text words. Similar searches were developed for each database, using appropriate controlled vocabulary and syntax. A detailed record is available in Supplementary Materials File S2.

Selection was conducted independently by two authors, with a third available for consultation and conflict resolution. All three authors were subjected to a study selection calibration exercise, on a batch of 50 random abstracts. Inter-researcher levels of agreement were estimated using Cohen’s kappa, with a target kappa value ≥ 0.70. Identified studies were centralised in a reference manager (Zotero v.7.0.32). Duplicates were identified and eliminated using the same software. Full texts of articles considered relevant (meeting the inclusion criteria) were obtained and evaluated independently. Conflicts were resolved by discussion and a third researcher’s input.

### 2.4. Data Extraction and Analysis

Data was extracted from included studies on pre-defined standardised forms designed for this study. Two reviewers extracted data independently. A third was available for supervision. The following data was extracted from the included clinical studies:

- General study characteristics (title, main authors, country of origin, DOI, year of publication, study design, total sample size, randomization method (where applicable), follow-up duration (in days/weeks))
- Population characteristics (age/gender distribution, psoriasis type and severity (expressed in psoriasis-related index points or percentages), periodontitis stage/grade, smoking status, comorbidities, psoriasis treatments)
- Intervention and comparator details (type of periodontal treatment, additional interventions, type of controls)
- Outcome-related data (PASI scores, QoL scores, periodontal outcomes (indexes expressed in percentage, millimetres or numeric values), systemic inflammatory marker outcomes (expressed in mean differences compared to baselines or control groups)
- For animal studies: animal characteristics (species, sex, age/weight, diet, disease induction method and model validation)

A minimum of 3 studies was considered for quantitative data synthesis; with clinically homogenous populations and interventions and low-to-moderate statistical heterogeneity (I^2^ < 75%). If criteria were not met, qualitative (narrative) synthesis of results was considered sufficient. Qualitative synthesis was presented in tabulated form.

### 2.5. Risk of Bias and Quality Assessment

Risk of bias and quality assessment was conducted by two reviewers working independently. We used the Cochrane Risk of Bias 2 (RoB 2) [24] Tool for the randomised clinical trials, the ROBINS-I tool (Risk Of Bias In Non-randomised Studies—of Interventions) [25] for the non-randomised trials, The Newcastle-Ottawa scale (NOS) [26] for cohort and case-control studies and the SYRCLE Risk of Bias Tool [27] for preclinical studies. Visualisations were generated using the robvis online tool [28].

## 3. Results

### 3.1. Study Selection

**Figure 2:**
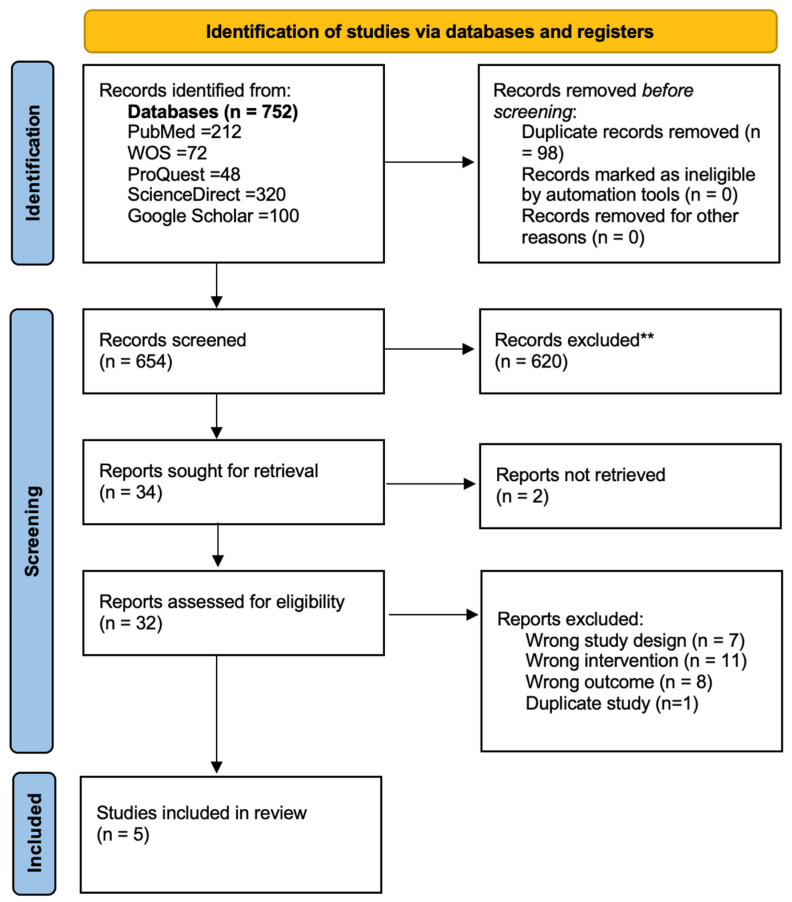
PRISMA 2020 flow diagram of study selection process.

The electronic literature search retrieved a total of 752 results. 32 studies were selected and retrieved in full-text form. Five studies were included in this review. Cohen’s kappa values were k = 0.79, indicating significant inter-researcher levels of agreement. The study selection process is illustrated in Figure 2.

### 3.2. Description of Included Studies

One preclinical study (murine model) was included. Out of the four clinical studies, two are randomised clinical trials, one study is non-randomised and one is a retrospective cohort study. General characteristics of included studies are summarised in Table 1. Due to the variety of study designs included, outcomes assessed in included studies and criteria for quantitative synthesis not being met, the results were qualitatively synthetised and presented. Despite two RCTs being included, both reporting PASI and periodontal parameters, we have decided against a limited quantitative synthesis, due to the following: different follow-up timepoints, different periodontitis stages at baseline, different number of treatment sessions.

Three studies directly assessed the influence of periodontal treatment on psoriasis outcomes in comorbidity situations. Two clinical studies by Marruganti et al. [13] and Ucan Yarkac et al. [29] assessed pre- and post-periodontal treatment outcomes by psoriasis-related outcomes (Psoriasis Area Severity Index (PASI) scores) and periodontal parameters as outcomes (probing depth—PD, clinical attachment loss—CAL, bleeding on probing—BOP, plaque index—PI, gingival recession—Rec, radiographic bone loss, etc.). These two studies investigated a total number of 166 patients. Marruganti et al. [13] additionally assessed the body surface area (BSA) affected by psoriasis and investigated the effects of psoriasis severity on patient’s quality of life, by the Dermatological Life Quality Index (DLQI). However, DLQI points decreased by 1.96 points, below the set MCIDs, demonstrating a non-statistically significant tendency towards quality-of-life improvement. Ucan Yarkac et al. [29] also assessed the inflammatory status of the patients, by salivary inflammatory markers (interleukins 2 and 6 and secretory immunoglobulin A (SIgA)). Both studies used non-surgical periodontal treatment interventions, on periodontitis cases diagnosed by the current consensus criteria. However, one study included only incipient and moderate periodontitis cases, while the other also included severe cases. The third study is a preclinical study by Marruganti et al. [30], in which the 56 male mice were divided into seven study groups, assessing the influence of periodontal treatment on psoriasis outcomes with and without systemic biologic agents (TNF- α inhibitors). Periodontal treatment was simulated by ligature removal, mimicking the effects of non-surgical periodontal treatment. All three studies acknowledged a statistically significant improvement in psoriasis-related outcomes post-periodontal treatment.

**Table 1:** Main characteristics of included studies.

Main Author, Year, Geographic Area	Study Design	Sample Size and Characteristics	Periodontal Treatment	Comparator	Outcomes	Results	Conclusions
CLINICAL STUDIES							
Marruganti et al. (2024). Italy [13]	RCCT	74 patients, comorbid periodontitis and psoriasis, age mean 57.90 (±12.1), 51 M (69.9%)/23 F, 1:1 test-control group allocation	Non-surgical with no antimicrobial agents (steps 1–2); oral hygiene instructions and motivational strategies, supra/subgingival instrumentation; 2–4 sessions	37 patients, delayed periodontal treatment	PASI + categorisation, BSA + categorisation; DLQI; periodontal parameters	10 weeks endpoint; statistically significant lower PASI (*p* = 0.001) and BSA scores (*p* < 0.05), and periodontal parameters for test group	In patients with both periodontitis and psoriasis, steps 1–2 of periodontal therapy showed an additional effect over conventional dermatological treatment in reducing the severity and extent of psoriasis.
Keller and Lin (2012). Taiwan [31]	Retrospective population-based cohort	230,730 patients (115,365 with periodontitis, 115,365 without periodontitis); 5 years follow-up for psoriasis incidence/progression; age mean 39.2 ± 16.2; 52.1% F	Surgical periodontal treatment: flap surgery and/or gingivectomy, within 1 year of diagnosis	Non-periodontitis cases	Risk of psoriasis development (HR)	5 years follow-up period; HR 1.26, (95% CI 1.01–1.60) for periodontal treatment; for non-periodontal treatment patients HR 1.55 (95% CI 1.40–1.76)	While treatment for periodontitis did attenuate the risk for subsequent psoriasis, it did not nullify the association between the two conditions.
Ucan Yarkac et al. (2019). Turkey [29]	RCCT	92 patients, comorbid periodontitis and psoriasis, 1:1 test-control group allocation	Non-surgical; oral hygiene instructions, subgingival/supragingival scaling and root planning; 4 sessions	46 patients, 8 weeks delayed periodontal treatment	PASI + categorisation; salivary IL-6, IL-2, secretory immunoglobulin A (sIgA); periodontal parameters	8 weeks endpoint; for test group; statistically significant decrease in IL-6 and IL-2 levels, PASI values, improvement in periodontal parameters (*p* < 0.05); statistically significant increase in sIgA levels (*p* < 0.05);	Effective periodontal therapy improves the psoriasis condition in patients afflicted by both diseases.
Ishihara et al. (2000). Japan [32]	NRCT	43 patients with comorbid palmoplantar pustulosis and periodontitis	Non-surgical and surgical (scaling, root planing or flap surgery)	11 patients, periodontitis only	Seric IgG antibodies against *E. coli* GroEL protein, synthetic *M. bovis* Hsp65 and recombinant Hsp60;	3 months endpoint; remission of PPP; significant reduction post-periodontal treatment of mean IgG levels in sera from PPP and periodontitis patients	Periodontal therapy and extraction of teeth with periapical infectious resulted in remission of pustulosis palmaris et plantaris and a statistically significant reduction in the levels of IgG against *E. coli* GroEL in 9 of the 22 patients (41%) examined.
PRE-CLINICAL STUDIES							
Marruganti et al. (2025). Italy [30]	Preclinical RCT	56 male mice, aged 8–12 weeks; 7 experimental groups: control group (P–Pso–) with no treatment; (b) periodontitis (P+Pso–) with periodontal therapy; (c) periodontitis (P+Pso–) with TNF-α inhibitor; (d) psoriasis(P–Pso+) with TNF-α inhibitor; (e) periodontitis and psoriasis (P+Pso+) with periodontal therapy; (f) P+Pso+ with TNF-α inhibitor; and (g) P+Pso+ with both periodontal therapy and TNF-α inhibitor	Periodontal treatment simulation—ligature removal after 14 days		Epidermal thickness and infiltrate cell [/0.03 mm^2^]; seric IL-6, IL-17A, TNF-α; distance between the cemento-enamel junction and alveolar bone crest [CEJ–ABC] and the number of osteoclasts	67-day endpoint; P+Pso+ group, a significant adjunctive effect of periodontal therapy to TNF-α inhibitors were found in the reduction in epidermal thickening and inflammatory infiltrate of the dorsal skin (*p* < 0.05), reduction in alveolar bone loss (*p* < 0.05), significant decrease in the circulating levels of IL- 6 and IL- 17A.	The combination of periodontal therapy and TNF-α inhibitor showed a positive synergetic effect in the treatment of comorbid experimental ligature- induced periodontitis and IMQ-induced psoriasis via the reduction in systemic inflammation.

Abbreviations: RCCT = randomised controlled clinical trial; NRCT = non-randomised clinical trial; RCT = randomised controlled trial; M = male; F = female; P = periodontitis; Pso = psoriasis; PASI = psoriasis area severity index; BSA = body surface area; DLQI = dermatological life quality index; HR = hazard ratio; IL = interleukin; sIgA = secretory immunoglobulin A; IgG = gamma-immunoglobulin; TNF-α = tumour necrosis factor-alpha; IMQ = imiquimod; CEJ–ABC = cemento-enamel-junction distance to alveolar-bone-crest; *E. coli* GroEL = chaperonin GroEL Escherichia coli; PPP = pustulosis palmaris et plantaris.

The study by Keller and Lin [31] indirectly assessed the influence of periodontal treatment on psoriasis; in a retrospective cohort study, the authors followed-up periodontitis and non-periodontitis patients to investigate psoriasis incidence and subsequent risk of psoriasis development in periodontitis patients, compared with non-periodontitis patients. They discerned lower hazard rates for psoriasis development in patients who received surgical periodontal treatment within one year of the diagnosis; only a slightly higher adjusted risk of psoriasis than the comparison patients was detected (HR 1.26, 95% CI 1.01–1.60).

The study by Ishihara et al. [32] was included although it is investigating palmoplantar pustulosis (PPP), a disease form whose classification has been widely debated as being either a subtype of psoriasis or a separate affliction. The reasons for inclusion are elaborated in the Discussions sections. This study compared seric antibody responses to heat-shock proteins, from patients with comorbid palmoplantar pustulosis and periodontitis compared to periodontitis-only patients. It concluded that periodontal treatment resulted in remission of PPP and a significantly reduced circulating IgG levels, suggesting remission of PPP by treating focal infectious sources. However, the findings of this study should be interpreted with caution in the context of the overall evidence presented in this review. PPP shares numerous manifestations with psoriasis variants, but presents significant differences as well, among which is the clinical association with focal infections. Therefore, it is expected that the clearance of focal infections (including through periodontal treatment) will reduce disease manifestations, for this particular form of dermatosis. These findings should be interpreted as more hypothesis-generating, rather than directly applicable to plaque psoriasis.

### 3.3. Risk of Bias and Quality Assessment

**Figure 3:**
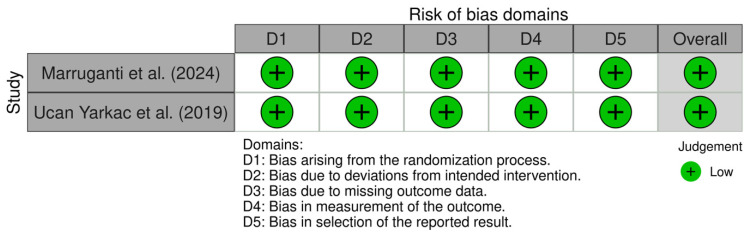
ROB-2 assessment for randomised clinical studies [13,29].

**Figure 4:**
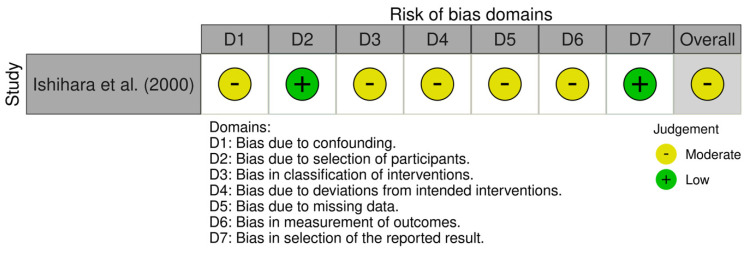
ROBINS-I assessment for non-randomised clinical studies [32].

**Figure 5:**
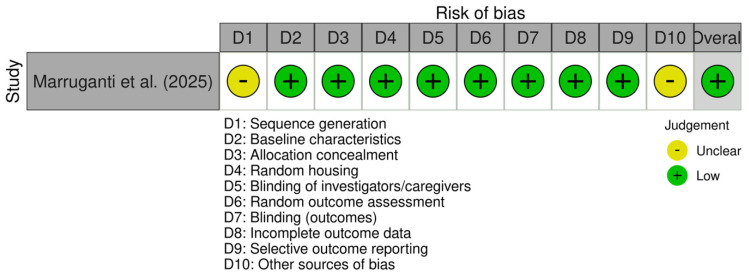
SYRCLE assessment for pre-clinical studies [30].

Both recent randomised clinical trials included were considered to have low risk of bias. Assessment results are available in Figure 3. The non-randomised study was considered to have moderate risk of bias due to one domain being impacted. Assessment results are available in Figure 4. The cohort study NOS assessment is available in Table 2. The pre-clinical study was considered to have low risk of bias. Assessment results are available in Figure 5. These results indicate a low risk of bias. However, the small number of studies and mixed designs reduce the certainty of evidence despite low risk of bias ratings. There are two RCTs reporting similar outcomes (PASI score changes in absolute points and periodontal parameters), for which we included a certainty-of-evidence framework assessment (GRADE) (Table 3). This was centred on PASI score changes, since the periodontal parameters for improvement post-periodontal treatment are very well established. The data pooled from two RCTs with a total number of 166 participants resulted in a low level of certainty of evidence, according to GRADE assessment. This suggests that in patients with comorbid periodontitis and psoriasis, periodontal treatment may reduce PASI scores by 2–4 points compared to control patients without periodontal treatment, assessed at 8–10 weeks. The direction of effect was consistent across both studies, despite different psoriasis severity populations and periodontal severity populations overlapping only partially.

## 4. Discussion

This systematic review summarised the existing evidence linking periodontal treatment to improved psoriasis outcomes. Despite being limited by a reduced number of studies currently available, the evidence proved to be qualitative, with risk of bias assessment revealing four studies with low risk of bias and one with moderate risk of bias. This provides new evidence into the bidirectional relation between periodontitis and psoriasis. Although being widely investigated, consensus has not been yet reached [14].

Preclinical studies with comorbid models of psoriasis and periodontitis are scarce; Marruganti et al. [33] developed a murine model with IMQ (imiquomod)-induced psoriasis and ligature-induced periodontitis and investigated the possible associations between the two pathologies, observing exacerbated periodontitis and psoriasis responses (epidermal thickening and inflammatory infiltrate) in the comorbidity group when compared to the non-periodontitis group, suggesting periodontitis could trigger psoriatiform inflammation. The same study suggested that psoriasis may elicit exaggerated periodontal manifestations as well [32]. This could potentially be explained by the Th17-axis activation in psoriasis, leading to increased seric cytokines and CRP levels. Increased cytokine levels promote recruitment and stimulation of other immune cells (e.g., neutrophils), establishing a positive feedback loop which is under-regulated in both periodontitis and psoriasis, thus possibly causing the onset of pathologies [33]. In a follow-up experiment, the same authors highlighted the adjuvant role of periodontal treatment in psoriasis outcomes, by itself and when combined with TNF-alpha inhibitors. The combined treatment approach showed a synergic effect on both pathologies, suggesting periodontal treatment could be successfully used in patients who are under systemic psoriasis management [29]. However, the preclinical study by Marruganti et al. [30] should be interpreted with caution, as it cannot fully reproduce the chronic characters of both diseases as encountered in humans, and cannot reproduce periodontal treatment fully. Aspects like systemic cytokine reduction and distance between the cemento-enamel junction and alveolar bone crest [CEJ–ABC] are likely translatable and reproductible in human studies, but effects like magnitude of PASI effect and interaction with biologic therapeutics remain largely speculative and are currently supported by limited evidence [13,29,31]. Despite this, a possible explanation for previous conclusions lies with systemic bacterial translocation. Dysbiosis and systemic bacterial dissemination characteristic to periodontitis may lead to bacterial translocation to the skin and gut microbiota; skin and gut microbiota have been demonstrated to influence each other, with skin dysbiosis being a possible trigger for psoriasis onset [34,35]. Furthermore, periodontitis onset and progression has been linked to dysbiotic gut microbiota; this highlights a possible organism-wide microbiological explanation for immune-mediated diseases, with potential future treatment targets [36]. Indeed, periodontal treatment has been demonstrated to modify gut microbiota composition [37,38,39]. Further pre-clinical studies and clinical follow-ups could potentially confirm these findings.

Three studies included in this review assessed the influence of periodontal treatment on inflammatory biomarkers in psoriasis patients [13,28,29]; all three studies investigated different molecules (Il-6, IL-2, sIgA, IgG, IL-17A, TNF-α), with only IL-6 being recurrent in two studies (one clinical and the other pre-clinical) [28,29]. Periodontal treatment has been proven to decrease cytokine levels in serum and saliva, and while the evidence provided by the included studies is not sufficient for quantitative analysis, it confirms previous findings [40,41] and suggests a systemic anti-inflammatory effect of periodontal treatment, supported by the decrease in all investigated molecules across studies. This may explain the results obtained by Keller and Lin [31], who observed a decreased risk of psoriasis development in patients who received periodontal treatment within one year of periodontitis diagnosis, compared with patients who were not treated for periodontitis. Furthermore, the decrease in severity of psoriasis lesions seems to derive from the same mechanism of inflammation reduction [13,28,29,42].

We included a study on palmoplantar pustulosis (PPP) in this review. PPP is considered a classification “grey zone”, as western guidelines and literature sometimes refer to PPP as palmoplantar pustular psoriasis, considered a particular form of pustular psoriasis [43]. Meanwhile, Japanese guidelines highlight the particularities of PPP as a separate disease entity [44]. The European ERASPEN consensus is that PPP is a variant of pustular psoriasis, which may be comorbid or not with psoriasis vulgaris [45]. Cited differences include epidemiological differences (with the Japanese population having a 15-fold higher prevalence [46]), possible genetic and environmental triggers, and strong clinical association with focal infections. Focal infection (including dental, periodontal and tonsillar) clearance usually results in complete remission of the episode and is integrated in Japanese treatment schemes [31,47,48,49]; this model of association is not replicated in western treatment, as pharmaceutical treatments are usually preferred [50,51]. However, psoriasis and PPP share phenotypes and inflammatory pathways, with increased levels of IL-8, IL-1α, IL-1β, IL-17A, IL-22, IL-23A present in PPP lesions. These shared inflammatory pathways extend to periodontitis, and therefore the proven anti-inflammatory effect of periodontal treatment on PPP may extend to psoriasis vulgaris (PV) as well, including in comorbid situations of PPP and PV. Excluding this study from this systematic review would neither strengthen or diminish measures of effect, as the study presents a different design compared to others included, and assesses different parameters as main outcomes. Including a PPP study was justified from a hypothesis-driven approach, due to similar physio pathological mechanisms of disease, and supportive mechanistic evidence provided; however, the findings of this study should be interpreted with caution as directly applicable only to PPP, and not as applicable to other forms of psoriasis directly, but rather as providing additional information on a still under-researched and not fully understood subject.

We acknowledge several limitations for this study, starting with the small number of included studies and evidence currently available. Furthermore, criteria for meta-analysis were not met, hence we could not provide a quantitative synthesis. Only three interventional studies directly assessed psoriasis outcomes after periodontal treatment, while another study mentioned remission of PPP and significantly decreased immunoglobulins post-treatment. Most studies presented small to medium endpoint periods, decreasing the strength of evidence by a lack of longitudinal follow-up. The reduced number of studies with homogenous population and interventions translated to a lack of quantitative data synthesis (meta-analysis). Furthermore, the psoriasis treatment of the study sample was inconsistently recorded, which may lead to confounding in results, due to systemic psoriasis medication having effects on periodontal tissues. The basal oral hygiene habits of study participants were insufficiently recorded, hence being a possible source of bias. Additionally, the preclinical study with IMQ-induced psoriasis and ligature-induced periodontitis models reflects an acute state of inflammation, unlike the chronic inflammation present in both diseases [29].

This review presents rigorous methodology, a protocol registered on PROSPERO and adherence to PRISMA 2020 guidelines; furthermore, transparent documentation of search strategies is available. To the authors’ knowledge, this is the first systematic review to assess available evidence linking periodontal treatment to improved psoriasis outcomes. This review presents some strengths adding weight to its conclusions and establishing causality: the inclusion of three high-quality RCCTs (two clinical and one preclinical) which show consistent findings and use validated outcomes. The two clinical RCCTs both investigate comparable interventions and show statistically significant effects of periodontal treatment on widely used psoriasis outcomes, like PASI. All included studies showed moderate to low levels of risk of bias. The inclusion of a high-quality pre-clinical study provides mechanistic validation, strengthening the conclusions of clinical studies and emphasising biological plausibility. Furthermore, the inclusion of a large-scale cohort study which links effects of periodontal treatment to lower risks for psoriasis development adds weight to the hypothesis. Additionally, the inclusion of an immunology study on a distinct disease form may support further studies assessing immune and inflammatory biomarkers and bridge knowledge gaps between psoriasis and PPP in relation to oral health and focal infections.

While mechanistic evidence continues to be revealed, the link between psoriasis and periodontitis may become more firmly established; this could reinforce the conclusions of this study, leading to further research being developed. Further studies should focus on investigating larger sample sizes with comorbid psoriasis and periodontitis, including all stages and grades of periodontal disease in the study sample and comparing inter-group therapeutic responses. Oral hygiene habits should be recorded and adherence to non-surgical periodontal treatment (which includes personal plaque control) should be estimated, as oral hygiene is a determinant in the rate of long-term periodontal treatment success. Psoriasis treatments (topical or systemic) should be considered in future studies, as to limit confounding and lead to biassed results. Furthermore, results could include pre-specified subgroup analyses stratified by biologics treatments (anti-TNF vs. anti-IL-17 vs. anti-IL-23). Longer follow-up periods (52 weeks) should be considered, in order to assess time to psoriasis relapse, efficacity of periodontal treatment and whether periodontal flare-ups may trigger further psoriasis development. Future studies could benefit from the integration of AI-based tools for psoriasis diagnosis (confirmed by a dermatologist) and further disease progression and PASI score monitoring, based on clinical images of the patients’ evolution before and after periodontal treatment. This could facilitate consistent longitudinal tracking across visits and facilitating correlation of image-derived improvement with periodontal parameters (PD/CAL/BOP) and inflammatory biomarkers (e.g., IL-6). LLMs (large language models) have been previously used successfully in dermatological diagnosis [52]. Another potential research focus should be the impact on quality of life and oral health-related quality of life of patients with comorbid pathologies. Furthermore, studies comparing patients with and without systemic treatment for psoriasis in addition to periodontal treatment, could provide new evidence to sustain or refine currently identified conclusions. Clinically, the interdisciplinary link between periodontitis management and psoriasis management could be used to elaborate future psoriasis management guides, highlighting a holistic approach in psoriasis treatment strategies and strengthening collaborations between dermatology and oral health professionals.

### Implications for Clinical Practice

Current existing evidence supports a bidirectional association between periodontitis and psoriasis but has not confirmed a causal relationship. However, periodontal assessment could be reasonably incorporated into the holistic approach of psoriasis, since both periodontal assessment and non-surgical treatment are minimally invasive and recommended as part of routine oral healthcare. Periodontal treatment could support the management of psoriasis management, mainly as part of comprehensive oral care and the management of potential adverse effects of antipsoriatic medication, rather than a confirmed adjunct treatment method. This approach may improve oral health and furthermore modulate systemic inflammation but cannot be suggested to patients as an adjunct therapeutic approach specifically for psoriasis management as of yet, due to limited evidence available. However, dermatologists and other specialists managing psoriasis could consider interdisciplinary collaboration with dental professionals for periodontal screening and treatment, especially for patients presenting with poor oral health status and moderate to severe periodontal symptoms, biologic therapies, and with increased psoriasis severity or affected surface areas.

## 5. Conclusions

Currently available evidence derived from a limited number of small-scale studies suggests periodontal treatment may offer a potential adjunctive effect on psoriasis outcomes like severity, affected body area and levels of inflammatory biomarkers. This could be explained by the interconnections of oral, skin and gut microbiota, along with shared inflammatory pathways. However, the current evidence level of certainty is low, limited by small sample sizes, short follow-up periods, variable periodontitis and psoriasis severity and indirectness due to mixed study designs. Further clinical studies with longer follow-ups and adequate controls are needed to consolidate these findings, while pre-clinical studies on experimental comorbid psoriasis and periodontitis models could elucidate the bidirectional association of these pathologies and provide further evidence supporting a potential causal interrelation.

## Figures and Tables

**Table 2:** NOS assessment [30].

Study	Selection				Comparability	Outcome			Total Score
	Representativeness of the exposed cohort	Selection of the non-exposed cohort	Ascertainment of exposure	Demonstration that outcome of interest was not present at start of study	Comparability of cohorts on the basis of the design or analysis	Assessment of outcome	Was followed up long enough for outcomes to occur	Adequacy of follow-up of cohorts	
Keller et al. (2012) [31]	*	*	*	*	**	*	*	**	10

“*” and “**” represent the number of points obtained for each section (1 and 2).

**Table 3:** GRADE certainty of evidence assessment for PASI scores changes.

Study Characteristics	Quality Assessment	Effect	Certainty
Design: RCTs Studies: Ucan Yarkac et al. (2019). Turkey [29], Marruganti et al. (2024). Italy [13] Participants: 166 (83 test group/83 control group) Follow-up: 8 to 10 weeks	Risk of bias: Serious (−1) Reason: Both studies lack patient blinding (not feasible in periodontal therapy trials) and have unblinded operators Inconsistency: Not serious Reason: Both studies show the same direction of effect: PASI decreased significantly more after periodontal therapy. Indirectness: Not serious Reason: Both studies enrolled adults with diagnosed periodontitis + psoriasis vulgaris Imprecision: Serious (−1) Reason: Small sample sizes in both studies, wide confidence intervals in Marruganti et al. [13]., PASI not primary outcome in Ucan Yarkac et al. [29].; not downgraded by 2 because both individually reached significance and the CIs exclude no effect. Publication bias: Not suspected Reason: No conflicts of interest determined; no industry funding; results sections accurately reflecting materials and methods sections; despite funnel plot assessment being impossible, both studies were registered in ClinicalTrials.gov before enrolment (NCT03939936; NCT05311501).	Control group: - Ucan Yarkac et al. [29]. MD not reported as such (estimated from data tables: MD = −0.36, 95% CI (−1.06, 0.34)); - Marruganti et al. [13]. MD +0.81, 95% CI (−1.24, 2.86) (reported as such) Intervention group: - Ucan Yarkac et al. [29]. MD not reported as such (estimated from data tables: MD = −2.34, 95% CI −3.36 to −1.32); - Marruganti et al. [13]. MD = −3.96, 95% CI −6.31 to −1.61 (reported as such) Interpretation: lower PASI indicates improvement in psoriasis symptoms; MD of −2.34 to −3.35 suggest moderate reduction in psoriasis severity.	Starting level: ⊕⊕⊕⊕ HIGH Downgrade: Risk of bias (−1) Imprecision (−1) FINAL: ⊕⊕◯◯ LOW

## Data Availability

Data sharing is not applicable to this article as no new data were created or analysed in this study.

## Supplementary Materials

The following supporting information can be downloaded at: https://www.mdpi.com/article/10.3390/jcm15062434/s1, File S1: PRISMA 2020 checklist; File S2: Search strategy.

## Abbreviations

The following abbreviations are used in this manuscript:

AAP/EFP	American Academy of Periodontology and European Federation of Periodontology
BOP	Bleeding on probing
BSA	Body surface area
CAL	C reactive protein
CRP	Clinical attachment loss
DLQI	Dermatological life quality index
IL	Interleukin
NRCT	Non-randomised clinical trial
PASI	Palmopalmar pustulosis
PD	Plaque index
PI	Probing depth
PPP	Psoriasis area severity index
PV	Psoriasis vulgaris
RCCT	Randomised clincal trial
RCT	Randomised controlled clinical trial
REC	Recession (gingival)
sIgA	Secretory immunoglobulin A
TNF	Tumour-necrosis-factor
