# Supplementary material for: The Effects of Periodontal Treatment on Psoriasis: A Systematic Review of Limited Clinical and Preclinical Evidence
Source: J Clin Med. 2026 Mar 22;15(6):2434. doi: 10.3390/jcm15062434 (PMC13028591; doi:10.3390/jcm15062434)
Supplement: Supplementary file 1 [file jcm-15-02434-s001.zip › jcm-4177566-supplementary.pdf]

Annex 1
SEARCH STRATEGY

CORE TERMS

Psoriasis terms
- "psoriasis"[MeSH Terms]
- Psoriatic
- Psoriasis [tiab]

Periodontitis/periodontal disease terms
- Periodontitis[MeSH]
- "gingivitis"[MeSH Terms]
- "gingiva"[MeSH Terms]
- "Periodontal diseases" [MeSH]
- Periodontitis[tw]
- **periodontal disease*[tiab]**
- **gingival disease*[tiab]**
- **"chronic periodont*"[tw]**
- **Periodontal pocket [tw]**
- Peri-implantitis

Periodontal treatment/intervention terms
- "periodontal therapy"[tw]
- "periodontal treatment"[tw]
- Scaling [tw]
- "root planing"[tw]
- "non-surgical periodontal"[tw]
- "periodontal debrid*"[tw]
- "Periodontal surgery"[tw]
- "flap debridement"[tw]
- "periodontal intervention"[tw]

Animal studies
- **Animals"[Mesh]**
- **Mouse**
- **Mice**
- **Murine**
- **Rat**
- **Rats**
- **Rodent**
- **Animal model**
- **Experimental**
- **In-vivo**
- **Ligature-induced**
- **Imiquimod**

- “ligature-induced periodont*”

PubMed

#1	"Psoriasis"[Mesh] OR psoriasis[tiab] OR psoriatic[tiab]	76,230
#2	periodontitis[MeSH] OR periodontitis OR "periodontal disease*"[tw] OR "periodontal infection*"[tiab] OR "chronic periodont*"[tw] OR "gingival disease*"[tw] OR gingivitis OR "gingiva"[MeSH Terms] OR "periodontal pocket*"[tw] OR peri-implantitis	182,026
#3	"Periodontics"[Mesh] OR "periodontal therapy"[tw] OR "periodontal treatment"[tw] OR "scaling"[tw] OR "root planing"[tw] OR "periodontal debridement"[tw] OR "non-surgical periodontal"[tw] OR "periodontal surgery"[tw] OR "flap surgery"[tw] OR "gingivectomy"[tw] OR "guided tissue regeneration"[tw] OR "periodontal intervention"[tw]	116,809
#4	#1 AND #2 AND #3	31
#5	**"Animals"[Mesh] OR mouse[tw] OR mice[tw] OR murine[tw] OR rat[tw] OR rats[tw] OR rodent*[tw] OR "animal model*"[tw] OR "experimental"[tw] OR preclinical[tiab] OR "in vivo"[tiab] OR "ligature-induced"[tw] OR "imiquimod"[tw] OR “ligature-induced periodont*”**	29,687,168
#6	#1 AND #2 AND #5	209
#7	#4 OR #6	212

Similar searches will be adapted for other databases using appropriate controlled vocabulary and syntax.

WOS

#1	**psoriasis OR psoriatic** (All Fields)	97,621
#2	**ALL=(periodontitis OR gingivitis OR peri-implantitis OR periodontal disease* OR chronic periodontitis)**	82,153
#3	**ALL=(periodontal therapy OR periodontal treatment OR "non-surgical periodontal" OR "flap debridement" OR "root planing" OR scaling OR "periodontal surger*" OR "root debridement")**	4,038,131
#4	#1 AND #2 AND #3	57
#5	**AB=(Animal* OR mouse OR mice OR murine OR rat OR rats OR rodent* OR "animal model*" OR "experimental" OR preclinical OR "in vivo" OR "ligature-induced" OR imiquimod OR “ligature-induced periodont*”)**	8,425,606
#6	#1 AND #2 AND #5	22
#7	#4 OR #6	72

ProQuest

summary(psoriasis) AND summary(periodontitis) AND summary(treatment) **48**

Google Scholar

Manual search by title/abstract for the first 100 results

Elsevier ScienceDirect

Psoriasis AND periodontitis AND treatment 320 results

Filters: Research articles
